# Genome-based development and clinical evaluation of a customized LAMP panel to rapidly detect, quantify, and determine antibiotic sensitivity of *Escherichia coli* in native urine samples from urological patients

**DOI:** 10.1007/s10096-024-05030-3

**Published:** 2025-01-07

**Authors:** Moritz Fritzenwanker, Marcel O. Grabitz, Vera Negwer, Oliver Schwengers, Borros Arneth, Trinad Chakraborty, Can Imirzalioglu, Florian Wagenlehner

**Affiliations:** 1 Institute of Medical Microbiology, Justus Liebig University Giessen, Giessen, Germany; 2 Bioinformatics and Systems Biology, Justus Liebig University Giessen, Giessen, Germany; 3 Clinic for Urology, Pediatric Urology and Andrology, Justus-Liebig-University, Giessen, Germany; 4 Institute of Laboratory Medicine and Pathobiochemistry, Phillips University, Marburg, Germany; 5 German Center for Infection Research (DZIF), partner site Giessen-Marburg-Langen, Giessen, Germany

**Keywords:** Urinary tract infections, Loop-mediated isothermal amplification, Antimicrobial stewardship, Point of care testing, Antibiotic susceptibility testing, Fast microbiology

## Abstract

**Purpose:**

We designed and tested a point of care test panel to detect *E.coli* and antibiotic susceptibility in urine samples from patients at the point of care in the urological department. The aim of this approach is to facilitate choosing an appropriate antibiotic for urinary tract infections (UTI) at first presentation in the context of increasing antibiotic resistance in uropathogens worldwide.

**Methods:**

We analyzed 162 *E.coli* isolates from samples from a university urological department to determine phenotypic and genotypic resistance data. With this data we created customized LAMP (loop-mediated isothermal amplification) panels for a commercial machine with which to detect and possibly quantify *E.coli* and six antibiotic resistance determinants. In a second step we tested these panel(s) for diagnostic accuracy on 1596 urine samples and compared with routine microbiological culture.

**Results:**

*E.coli* was detected with 95.4% sensitivity and 96.1% specificity. Dynamics of the LAMP amplification could be used to gauge bacterial loads in the samples. Antibiotic sensitivity was detected with good negative (sensitive) predictive values: ampicillin 92.8%, ampicillin/sulbactam 96.4%, cefuroxime 92.8%, cefotaxime 97.8%, trimethoprim/sulfamethoxazole 96.5%, ciprofloxacin 96.8%.

**Conclusion:**

The LAMP panel provided *E.coli* detection and sensitivity information within one hour and thus could principally guide initial antibiotic therapy upon patients presenting with UTI. The panel helps to select initial adequate antibiotic therapy as well as providing diagnostic stewardship. Follow up investigations will expand the test system to other uropathogens.

**Supplementary Information:**

The online version contains supplementary material available at 10.1007/s10096-024-05030-3.

## Introduction

Urinary tract infections (UTI) are among the most common bacterial infections, with a globally rising trend in terms of burden of disease [1]. They are especially important in urology, as urologic complicating factors (such as urine stones, urinary catheters etc.) increase the risk for infections. Typically the most common uropathogen in UTIs by far is *Escherichia coli*, although *c*omplicated UTIs are associated with a more diversified spectrum and a higher risk of antimicrobial resistance [2–4]. The emergence of various resistances, especially in *E. coli* and other Enterobacterales has made it difficult to choose an effective antibiotic when a patient presents with symptoms. EAU (European Association of Urology) guidelines recommend prescribing only antibiotics for empirical therapy against which less than 10% of the local bacterial population is resistant. For uncomplicated cystitis, these are currently fosfomycin, nitrofurantoin and pivmecillinam which typically show very low resistance rates, but are not suitable for complicated UTIs. Previously, ampicillin, ampicillin-sulbactam, trimethoprim, trimethoprim-sulfamethoxazole and ciprofloxacin were considered primary choices for UTIs including complicated UTI, but in recent years /decades resistance rates increased to more than 10% in many uropathogens, making them unfavorable for empirical first line therapy. In their stead, physicians now need to prescribe antibiotics which were previously considered as “reserve”, such as third generation cephalosporines, aminoglycosides, piperacillin/tazobactam, or even carbapenems. However, resistance rates against these antibiotics are also increasingly becoming a problem. New substances are only slowly entering the market, making adequate initial empiric therapy more difficult. To preserve the effectiveness of the reserve antibiotics, antimicrobial stewardship (AMS) efforts recommend restricted use. In fact, the older, narrow-spectrum antibiotics can be quite as effective as the broad-spectrum ones, for as long as the bacteria that cause the infection are not of the resistant phenotype [5].

Some of these, namely ampicillin-sulbactam, trimethoprim-sulfamethoxazole and ciprofloxacin have desirable pharmacological features compared to the reserve antibiotics as they can be administered orally and are highly active in urine (allowing outpatient care) and are comparably cheap.

Despite overall resistance rates above 10%, there is typically a substantial part of the *E.coli* population that is susceptible to at least one of these antibiotics. However, physicians need to wait for at least 48 h to know which antibiotic shows in vitro effectivity, when relying on current gold standard microbiological culture with antibiotic susceptibility testing (AST) [5–10].

AMS recommends developing and deploying “fast microbiology” test systems, preferably at the point of care. The idea is that samples are tested during first patient visit, for the presence of bacteria and bacterial resistance. The initial antibiotic therapy is then to be guided by that information: the tests shall inform the physician which antibiotic should be avoided for their patients (due to resistance), and, in the absence of resistance, which patient can safely be treated with older, narrow-spectrum antibiotics.

Some approaches to this idea have been made in recent years, but to our knowledge none provides identification, quantification and AST within a few hours [11–19]. We conducted a proof-of-principle pilot study to design and evaluate a novel test system at the point of care. We chose LAMP (loop-mediated isothermal amplification) as basic technique, as it can rapidly and robustly detect genes (including resistance genes) in urine samples. Additionally, ready-to-use LAMP machines are on the market, and it is possible to generate custom testing panels for them, by designing primers targeting the desired genes, similar as with PCR (polymerase-chain-reaction).

**Fig. 1:**
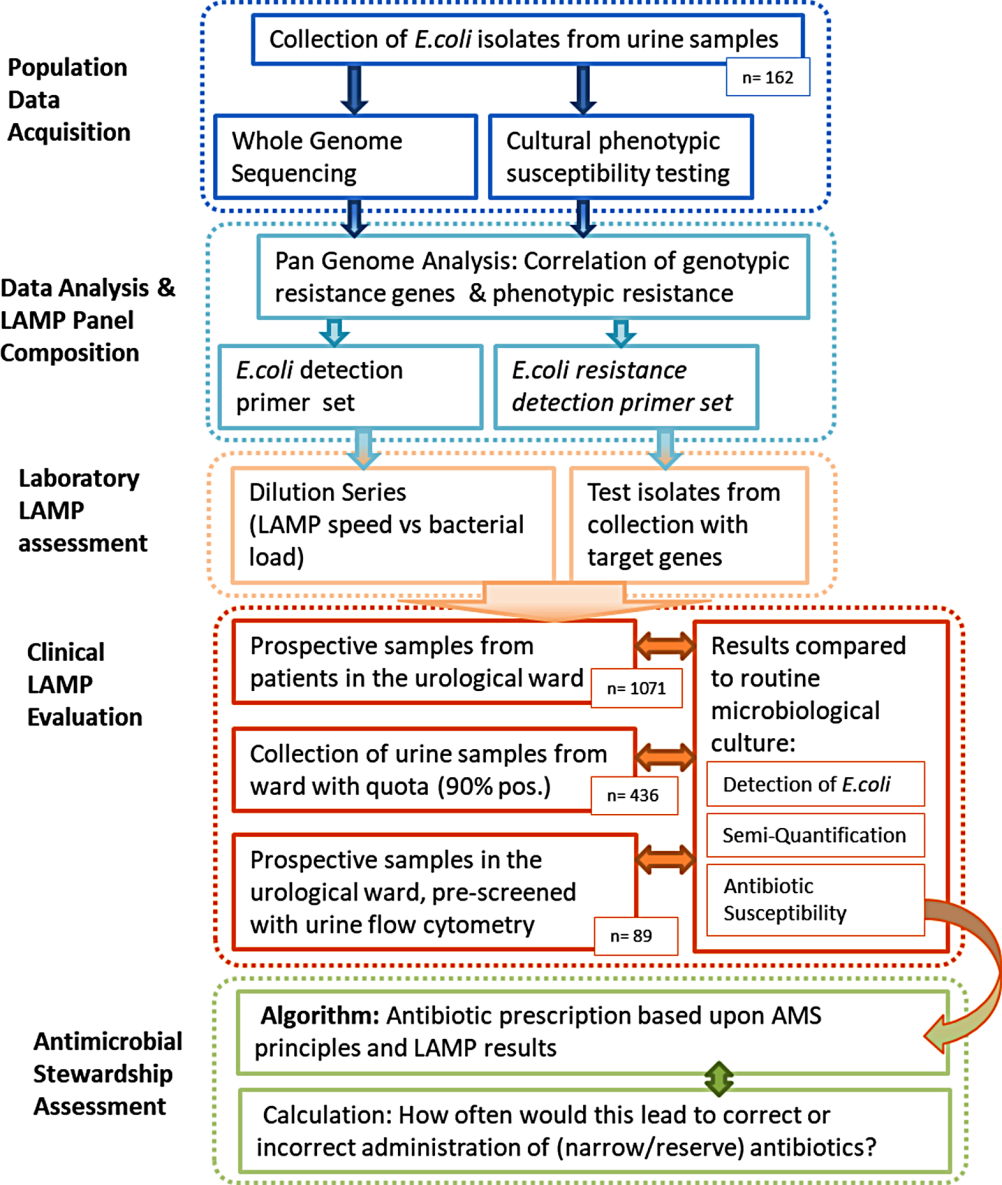
Project strategy

Our pilot study was conducted with the following aims (see Fig. 1 for the project strategy):

1. Design a customized LAMP panel for a commercially available system that can detect clinically relevant amounts of *E.coli* in urine samples.
2. Design an accompanying LAMP panel that subsequently detects antibiotic resistance or susceptibility in these samples with *E.coli*.
3. Test such panels prospectively at the point of care in a urological department, to assess whether useful microbiological results can be produced in a reasonable timeframe. These results would be compared with routine microbiological culture as the current state of the art.
4. Calculate how prescriptions based on LAMP results could improve antibiotic prescriptions with regard to AMS recommendations.

## Methods

### LAMP panel design

To find genetic markers for *E.coli* resistance we first collected 162 consecutive isolates from urine samples of the target population, which were patients of the university urological department (in-and outpatients). *E.coli* isolates were characterized with phenotypical methods (resistance testing on the Vitek MS 2 system (AST N264 cards, Biomerieux, Nürtingen, Germany) and subsequently sequenced on an illumina NextSeq with a NextSeq500 High Output flow cell using Nextera XT libraries (Illumina, the Netherlands). Genome sequences were assembled and analyzed using the in-house bioinformatics pipelines ASA [3]P, EDGAR, and Resfinder [20–22]. We compared phenotypic data to genome-based data to find the resistance genes or surrogate genomic markers most commonly associated with phenotypic resistance. We designed LAMP primers for genes of interest using PrimerExplorer (https://primerexplorer.jp/e/). Primer sets were then tested for functionality on isolates of this strain collection in vitro.

### LAMP reagents and instrumentation

The isothermal mastermix was purchased from Amplex (Gars, Germany). Oligonucleotide primers were purchased from Eurofins Genomics (Ebersberg, Germany). Culture media were from ThermoFisher Scientific (Waltham, USA).

### Patient population for LAMP panel evaluation

For the validation and evaluation study we tested three sets of urine samples from patients of the urological department:

1. Prospective urine samples, directly at the point of care in the urological department (*n* = 1071).
2. Urine samples collected at the department and stored for later analysis; samples were chosen randomly from the collection but with a quota of 10% culture-negative samples (this approach was intended to test whether LAMP would correctly identify *E.coli* within samples in which other species were present as well, and to examine whether species other than *E.coli* would be falsely detected, therefore the artificially low amount of negative samples; *n* = 436).

3) Prospective urine samples were tested again at the point of care in the urological department, but this time samples were pre-screened with urine flow cytometry (UFC; the idea being to use UFC to discard sterile urine samples, thus saving LAMP tests; *n* = 89). The UFC screening process was established previously, for details please see [23].

In total 1596 samples were analyzed, 1144 from male and 452 from female patients. 1247 patients were in outpatient care while 349 were inpatient admissions. There were no formal exclusion criteria for patients. Patients were diagnosed due to symptoms (none, local, local & systemic) and cultural findings of bacteria in their urine samples, according to EAU guidelines for urological infections [5].

### Workflow

**Fig. 2:**
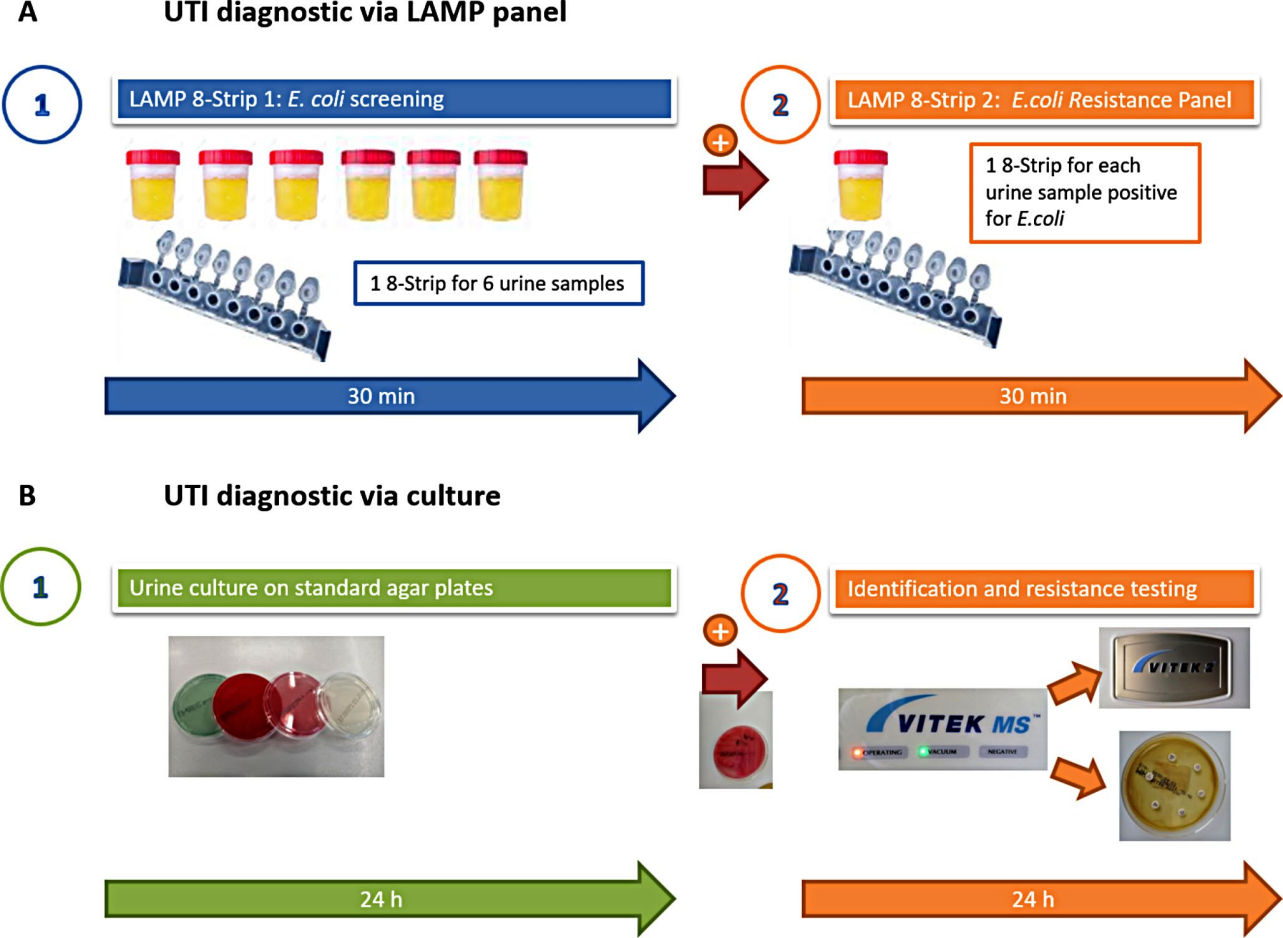
Diagnostic Workflow: **A** Urine samples are screened for *E. coli* with LAMP reactions. Positive samples are then tested for specific resistance genes. **B** The same urine samples are analyzed in the microbiological laboratory using standard urine culture and phenotypic susceptibility testing

The analysis workflow is shown in Fig. 2. Following sample acquisition we screened samples for the presence of *E.coli*, using the LAMP assay to detect *E. coli* specific *gyrA* gene. The LAMP machine uses 8-strips, allowing 8 LAMP reactions per run. For the first step of the test workflow, we filled 6 wells with identical primer sets, using each 8-strip to test up to 6 different urine samples. Two wells were used for negative and for amplification control.

Positive samples were subsequently tested using the antibiotic resistance gene panel. This is a second 8-strip with 6 different primer sets for *E.coli* resistance genes and one well for an amplification control (*gyrA* gene from above):

1. *blaTEM1-β*-gene as a representative of a narrow spectrum beta-lactamase.
2. *blaCTX-M-14* & *blaCTX-M-15* as representatives of extended spectrum beta-lactamases.
3. *sul2* & *dfr-A17* as indicators for resistance against trimethoprim/sulfamethoxazole.
4. *hlyA* gene as a proxy parameter for resistance to fluoroquinolones (see results and discussion for why we chose *hlyA*).

### LAMP reaction

1.5 ml urine sample were centrifuged (6 min, 5600 rpm), supernatant fluid was discarded, and the pellet re-suspended in 120 µl ddH_2_O. This was briefly vortexed and heated at 99 °C for 12 min. 5 µl of this sample solution was then mixed with prepared LAMP reaction mixture, which contained 15 µL isothermal Mastermix (Amplex; GspSSD -polymerase and nucleotides) with 1.6µM of FIP and BIP primers each, 0.4µM of F3 and B3 each, and 0.8µM LoopF and LoopR each (where applicable, not all sets had two loop primers), filled up with ddH_2_O to a total of 25 µl. PCR amplicon of *E. coli gyrA* (conc. 42.7ng/µl) and ddH_2_O served as positive and negative controls, respectively. Primer sets are given in supplemental Table 1.

Amplification was performed on a Genie II instrument (Optigen (UK), distributed by Amplex, Germany) using a temperature of 63 °C for 30 min followed by an annealing period of 2 min with a declining temperature from 98 °C to 80 °C. Samples were deemed as positive if the amplification exceeded 10,000 fluorescent units (FU) on the Genie II system graphical user interface.

### Controls

Our experimental data was measured against results provided by the routine diagnostics of the departments of microbiology, urology and clinical laboratory medicine.

Patients were initially screened using Uricult^®^Plus dipslide. Aliquots of each sample were sent directly to the microbiological laboratory for cultivation and data subsequently compared to that from the LAMP results. Where such aliquots were not available, LAMP data was compared to colony numbers detected on a dipslide sample. Aliquots of each sample were sent directly to the microbiological laboratory for cultivation and compared to the LAMP results (in cases without such aliquots, LAMP data was compared against dipslide data).

Urine samples were cultivated on CLED-Agar, CNA-Agar, MacConkey-Agar and Sabouraud-Agar (Thermo Fisher). Isolates were identified by Matrix Assisted Laser Desorption Ionization — Time of Flight Mass Spectrometry (MALDI-TOF MS)(VITEK MS, Biomerieux), followed by disc-diffusion and automated antimicrobial susceptibility testing (VITEK 2, Biomerieux), using EUCAST breakpoint criteria. Additional data using urine flow cytometry testing on a UF-1000i system (Sysmex, Kobe, Japan) was also incorporated into the analysis. LAMP assays and microbiological cultivation were performed independently from each other, in a blinded fashion, by different people who had no knowledge of the results of the respective other method at the time they analyzed the samples.

Statistical were calculated in Microsoft Excel, confidence intervals on MedCalc Software Ltd. Diagnostic test evaluation calculator (https://www.medcalc.org/calc/diagnostic_test.php).

### Semi-quantification test

Quantification of uropathogens is one factor in differentiating significant bacteriuria from contamination. Commonly used cutoffs for this are 10^5^ CFU/ml and 10^4^ CFU/ml. Detection using LAMP amplification is dependent on the concentration of template DNA in the sample. Thus, the time point at which an amplification curve reaches a certain threshold could be used to determine bacterial loads (analogous to the Cp and Ct values used in realtime-PCR). To test this hypothesis, we made four serial dilutions series of *E. coli* in ddH_2_O, tested these with LAMP reactions and plated each dilution out in parallel, and counted the colonies to determine CFU/ml. Time to positivity was defined as the time point at which the LAMP amplification curve reached 10,000 FU (fluorescence units, as displayed on the Genie II instrument). The data were analyzed in Microsoft Excel Plus 2016, with the Add-In XLMiner Analysis ToolPak by Frontline Systems.

## Results

### Design of a rapid, targeted genetic screening panel

We aimed to rule out resistance against specified antibiotics (high negative predictive value in comparison to cultural testing). Our goal was to predict sensitivity to antibiotics with at least 90% predictive value, to meet a cutoff proposed by the EAU guidelines for the calculated treatment of UTIs [24].

We first determined whether screening for a dominant clonal lineage of *E.coli* would allow antimicrobial resistance determination. Our analysis of the 162 *E.coli* isolates revealed that the only predominant sequence type was ST-131 (19%). Every other sequence type was present in lower frequency (i.e. ST-69 and ST-73 with 6% each, and many STs were represented by single isolates (see supplemental Fig. 1) [25]. Since ST-131 is a well-known emerging multinational clonal lineage, disseminating quinolone and beta-lactam resistance all over the world, we expected that detecting ST-131 might help to predict phenotypic resistance. However, a majority of the ST-131 harbored no notable antimicrobial resistance, and other STs contributed to the resistances as well (see supplemental Fig. 2). We examined the pan genome of the 162 isolates, to identify genes most closely associated with phenotypic antibiotic resistance. For each gene in the pan genome, we calculated how many isolates with that gene, and without that gene, were susceptible or resistant to each antibiotic of interest, to create sort of a four-field-board for each combination of gene and antibiotic. For each antibiotic, we looked at the candidate genes most closely associated with resistance. For most antibiotics, we found promising LAMP targets in the sense of antibiotic resistance genes that were present in most resistant strains, and absent in most susceptible strains, meaning we could theoretically expect high negative predictive values when used as screening parameter in the LAMP assay (see supplemental Tables 2 and 3 for details).

We identified common beta-lactamase genes (*bla*_TEM−1B_, *bla*_CTX−M−14_ and *bla*_CTX−M−15_) as feasible predictors for resistance against commonly used beta lactam antibiotics (ampicillin/sulbactam, cefuroxime and cefotaxime, see supplementary Fig. 3A).

In order to maximize the likelihood of predicting a sensitive phenotype, we chose both *bla*_CTX−M−14_ and *bla*_CTX−M−15_ as screening targets to exclude ESBL (extended spectrum β-lactamase) phenotype (i.e. isolates would be marked “sensitive” to cefotaxime when neither gene was detected) and *bla*_TEM−1B_ was chosen as denominator for resistance against ampicillin/sulbactam and cefuroxime. Not all isolates that carried *bla*_TEM−1B_ were phenotypically resistant to these antibiotics, but its absence clearly indicated sensitivity.

Similarly, we identified known genes in folate synthesis (mostly *sul1*, *sul2* and *dfrA17)* to be commonly present in isolates resistant to trimethoprim/sulfamethoxazole, suggesting these genes as prime candidates for resistance screening via LAMP amplification (see supplementary Fig. 3B). We chose to include one gene each for the dihydropteroate synthetase and dihydrofolate reductase steps in the folate pathway, in order to maximize the reliability of excluding phenotypic resistance when running the test.

Resistance against quinolones was not mediated by presence of a specific gene in the pan genome, but rather by commonly known mutations in the *gyrA* gene. While statistical correlation of mutation and resistance was 100%, we failed to produce LAMP primer sets that could reliably distinguish mutated *gyrA* from wild type in LAMP reactions. Genomic analysis suggested a potential proxy parameter: a *hlyA* gene of ≥ 2400 bp was associated with sensitivity (of 39 isolates, 35 were sensitive and 4 resistant), while isolates with no *hlyA* locus or supposedly truncated loci (< 2400 bp) displayed a higher rate of resistance (from 123 such isolates, 75 were sensitive and 48 resistant) (see supplemental Table 4). We proceeded to design the LAMP panel with “full length *hlyA*” as proxy parameter for fluoroquinolone sensitivity.

We validated the LAMP primer sets by testing them in vitro on isolates of the 162 fully sequenced *E. coli* isolates and then screened prospectively collected urine samples for the presence of *E.coli*, and subsequently for antibiotic resistance if *E. coli* was detected.

### Detection of *E. coli* by LAMP Screening

After designing the LAMP-panel we tested it with urine samples from patients of the urological department in three steps:

1. We tested prospective samples directly at the point of care: 96.1% sensitivity, 96.38% specificity.
2. We tested a randomized selection of stored samples with a quota of 90% culture-positive (any species) and 10% culture-negative urine samples: 94.7% sensitivity, 95.3% specificity.
3. We tested prospective samples at the point of care which were previously tested positive for bacteriuria with urine flow cytometry: 96.6% sensitivity, 95.2% specificity.

In total we tested 1596 urine samples of 1317 clinical cases (due to multiple samples from a subset of patients). Table 1 and 2shows diagnoses and urologically relevant secondary diseases of the patients.

**Table 1:** Diagnoses of patients whose urine samples were screened using the LAMP panel. The LAMP screening yielded the following results: 207 true positive, 1325 true negative, 10 false negative and 54 false positive. This yielded 95.39% sensitivity, 96.08% specificity overall, with a PPV of 79.31% and a NPV of 99.25%. We then analyzed subgroups of sex, presence of indwelling catheters and as outpatient/inpatient, but detected no notable differences. Importantly, sensitivity, and likewise the NPV were high in all groups, thus suggesting the test can safely be used to rule out *E.coli* in urine samples (see table 2)

Diagnosis	Cases (*n* = 1317)
**Infectious diseases**	
Uncomplicated lower UTI	16
Complicated lower UTI	164
Pyelonephritis	28
Kidney abscess	2
Urosepsis	8
Prostatitis	43
Orchitis/Epididymitis	31
Sexually transmitted diseases	11
**Macrohematuria**	66
**Urolithiasis**	162
**Tumors**	
Kidney tumor	58
Prostate carcinoma	103
Urothel carcinoma of the bladder	193
Urothel carcinoma of the upper urinary tract	18
Testicular tumor	11
Penile carcinoma	3
Benign prostate hyperplasia	96
**Others**	
Incontinence	75
Phimosis	16
Urinary tract strictures	151
Urinary bladder diverticulum	3
Bladder fistula	5
Kidney malformations	5
Urogenital tract injuries	14
Foreign bodies in the urinary tract	2

**Table 2:** Screening urine samples for *E.coli* with our LAMP panel. As compared to urine culture, 95%-confidence-intervals in brackets

	Sensitivity [%]	Specificity [%]	PPV [%]	NPV [%]	Accuracy [%]	*N*
All samples	95.39 [91.69, 97.77]	96.08 [94.92, 97.04]	79.31 [74.66, 83.30]	99.25 [98.64, 99.59]	95.99 [94.91, 96.90]	1596
Set 1: prospective urine samples, without pre-selection	96.10 [89.03, 99.19]	96.38 [95.02, 97.45]	67.27 [59.79, 73.97]	99.69 [99.06, 99.90]	96.36 [95.06, 97.40]	1071
Set 2: randomized selection of urine samples (quota 90% culture-positive with any species, 10% sterile)	94.74 [88.90, 98.04]	95.34 [92.43, 97.37]	87.80 [81.43, 92.20]	98.08 [95.91, 99.11]	95.18 [92.73, 96.99]	436
Set 3: prospective urine samples, pre-screened with urine flow cytometry	96.15 [80.36, 99.90]	95.24 [86.71, 99.01]	89.29 [73.37, 96.18]	98.36 [89.77, 99.76]	95.51 [88.89, 98.76]	89
Female	95.87 [90.62, 98.64]	96.37 [93.75, 98.11]	90.63 [84.71, 94.40]	98.46 [96.43, 99.34]	96.24 [94.05, 97.79]	452
Male	94.79 [88.26, 98.29]	95.99 [94.62, 97.10]	68.42 [61.61, 74.52]	99.51 [98.85, 99.79]	95.89 [94.57, 96.97]	1144
Indwelling catheter	95.06 [87.84, 98.64]	94.74 [89.46, 97.86]	91.67 [84.23, 95.77]	96.92 [92.37, 98.79]	94.86 [90.99, 97.41]	214
Disposable catheter	97.06 [84.67, 99.93]	97.33 [93.87, 99.13]	86.84 [73.50, 94.01]	99.45 [96.35, 99.92]	97.29 [94.18, 99.00]	221
Outpatient	95.32 [90.99, 97.96]	96.10 [94.76, 97.17]	79.51 [74.23, 83.95]	99.23 [98.50, 99.61]	95.99 [94.75, 97.01]	1247
Inpatient	95.65 [85.16, 99.47]	96.04 [93.18, 97.94]	78.57 [67.73, 86.50]	99.32 [97.40, 99.82]	95.99 [93.36, 97.79]	349

### Semi-quantification of *E. coli* CFUs using time-to positivity

**Fig. 3:**
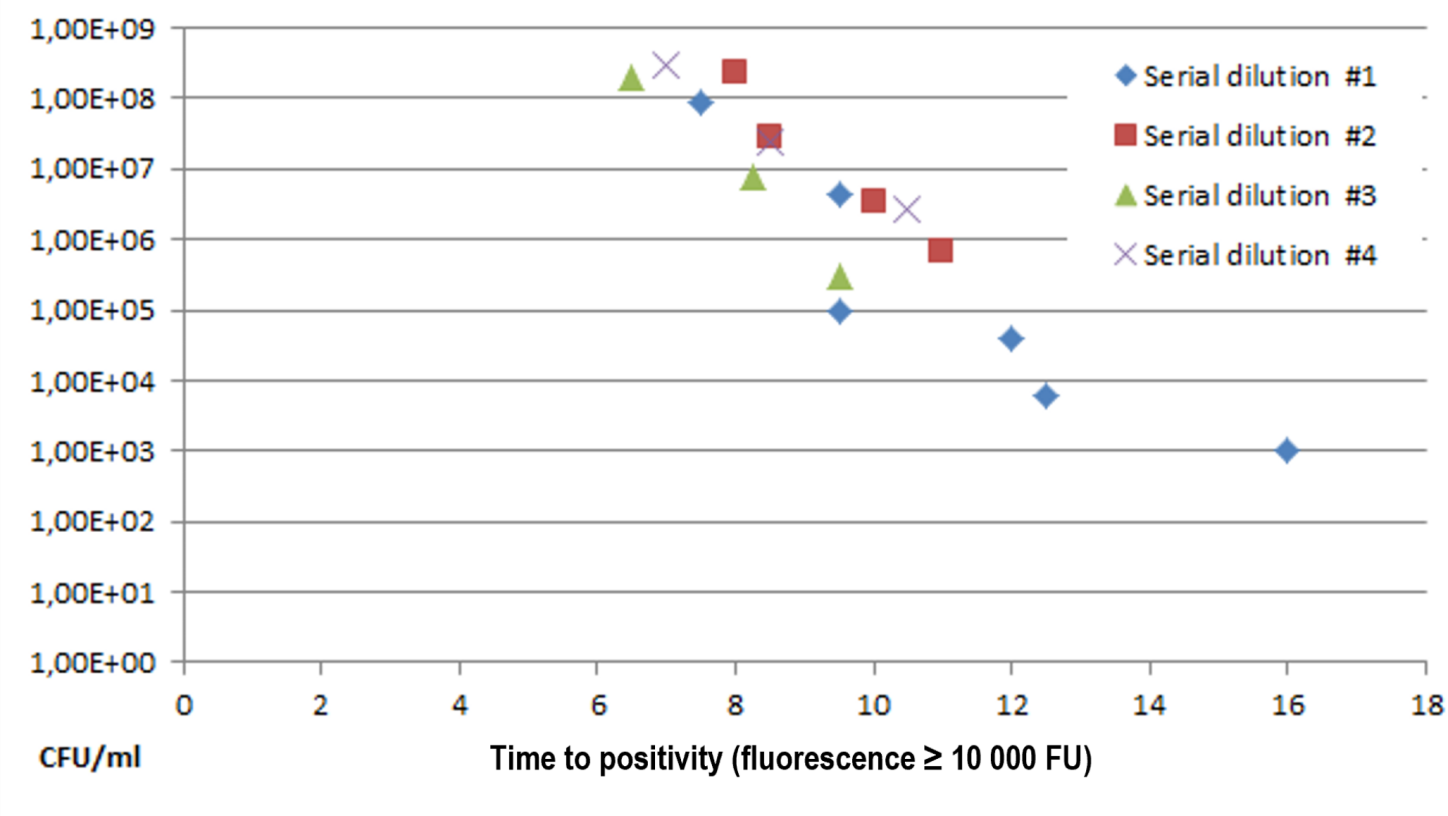
LAMP detection of *E.coli in vitro*, dilution series: *E.coli* was diluted and measured with LAMP. Higher *E.coli* concentrations mean that LAMP turns positive faster

To estimate bacterial loads we looked at Time-to-positivity of the LAMP amplification curves (“Cp values”). In vitro, four dilution series showed good correlation of time-to-positivity with CFU/ml (see Fig. 3): a regression analysis (linear regression with logarithmized values for CFU/ml) yielded: multiple R 0.92, R square 0.84, F 76.27 and significance of F 4.8621E-07.

We then determined whether this was true for urine samples from patients as well. Our routine laboratory stratifies bacterial loads into the following categories:

1. sterile.
2. ≤ 1 × 10³ CFU/ml.
3. 1 × 10³-1 × 10^4^CFU/ml.
4. 1 × 10^4^-5 × 10^4^CFU/ml.
5. 5 × 10^4^-1 × 10^5^CFU/ml.
6. > 1 × 10^5^ CFU/ml.

**Fig. 4:**
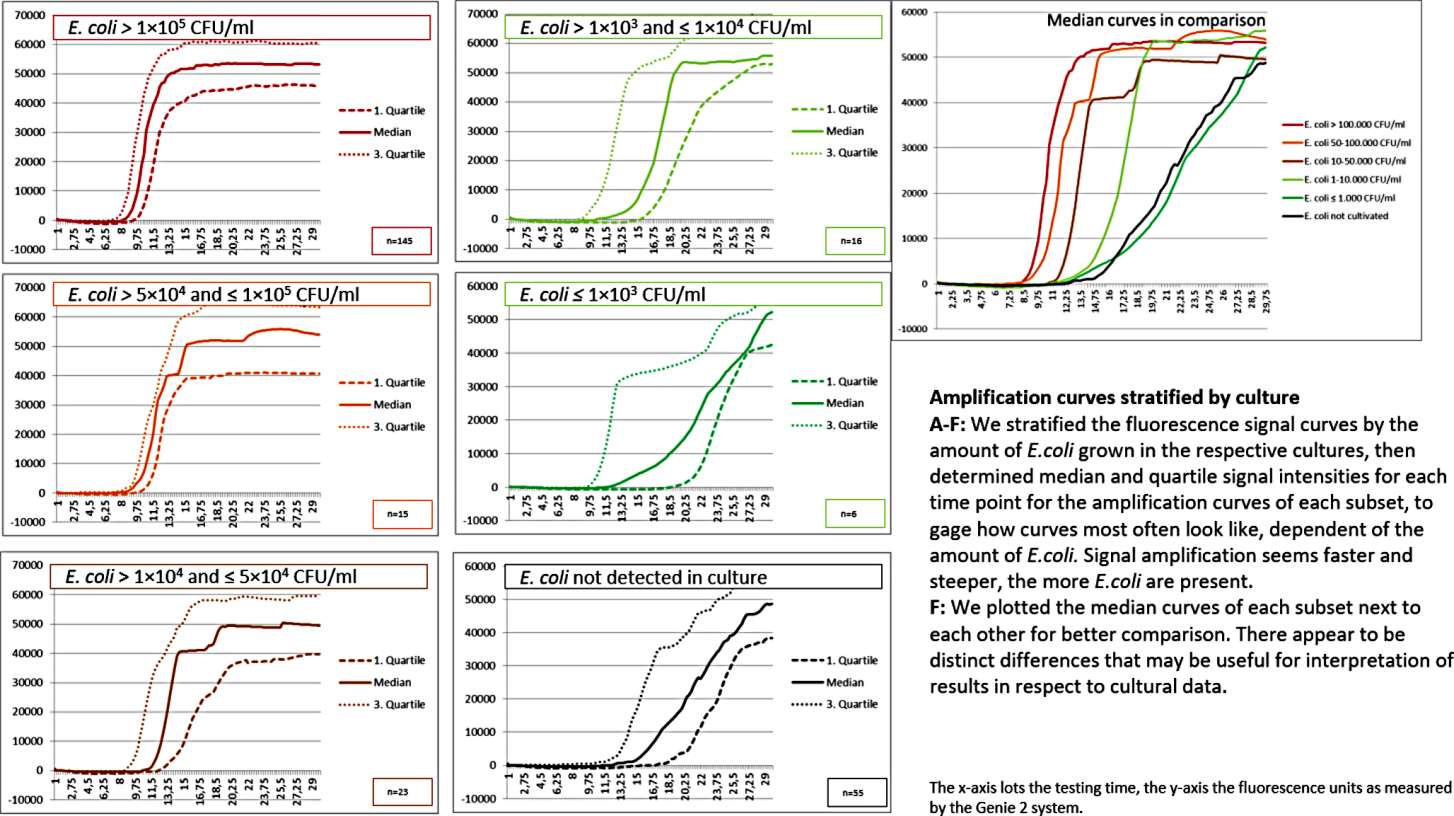
LAMP amplification curves from point of care testing of urine samples Sensitivity Forecast by LAMP-Panel

We clustered the LAMP amplification curves for each of these cultural load subgroups, and calculated arrays of curves for each, respectively (see Fig. 4). For each subgroup, we determined the median fluorescence signals at each time point to visualize the “typical” amplification curve for the respective bacterial load, and the first and third quartile to estimate the variation therein. As in our in vitro experiments, the onset of the amplification appeared to be dependent on the bacterial load in the samples, with higher loads meaning faster LAMP curves and vice versa. This was especially true in the subgroups with 1 × 10^4^ CFU/ml and higher – the subgroups with lower loads show more variation. The curves from samples with no cultivable *E.coli* also amplify very slowly, and thus can for the most part be distinguished from those with relevant bacteriuria.

Samples screened positive for *E.coli* with LAMP were then tested with the second panel for genes indicative of antibiotic resistance. We compared these data with the phenotypic resistance testing that was performed in the routine diagnostic workflow (see Table 3).

**Table 3:** Accuracy of LAMP resistance forecast, compared, routine microbiological testing. PPV in this case means that LAMP correctly predicts phenotypic resistance, while NPV means that LAMP correctly predicts phenotypic sensitivity. 95-confidence-intervals in brackets

Antibiotic	Sensitivity [%]	Specificity [%]	PPV [%]	NPV [%]	Accuracy [%]	*N*
ampicillin	92.0 [83.40, 97.01]	90.6 [82.29, 95.85]	89.6 [81.63, 94.36]	92.8 [85.59, 96.52]	91.3 [85.75, 95.13]	160
ampicillin/ sulbactam	94.9 [85.85, 98.94]	79.2 [69.99, 86.64]	72.7 [64.47, 79.67]	96.4 [89.81, 98.78]	85.0 [78.51, 90.15]	160
cefuroxime	76.9 [56.35, 91.03]	57.5 [48.63, 65.96]	26.0 [20.82, 31.88]	92.8 [86.24, 96.33]	60.6 [52.60, 68.25]	160
cefotaxime	83.3 [58.58, 96.42]	95.1 [90.11, 98.00]	68.2 [50.27, 81.96]	97.8 [94.12, 99.22]	93.8 [88.81, 96.96]	160
piperacillin/ tazobactam	70.0 [34.75, 93.33]	90.1 [84.15, 94.33]	31.8 [19.93, 46.67]	97.8 [94.61, 99.15]	88.8 [82.91, 93.24]	161
ceftazidime	76.9 [46.19, 94.96]	91.9 [86.27, 95.74]	45.5 [30.98, 60.74]	97.8 [94.38, 99.19]	90.7 [85.10, 94.69]	161
trimethoprime/ sulfamethoxazole	90.7 [77.86, 97.41]	92.4 [86.01, 96.45]	81.3 [69.66, 89.11]	96.5 [91.45, 98.58]	91.9 [86.59, 95.63]	161
ciprofloxacin	97.2 [85.47, 99.93]	24.0 [16.82, 32.46]	26.9 [24.76, 29.20]	96.8 [80.90, 99.53]	40.4 [32.72, 48.38]	161

### AMS benefit calculation

**Fig. 5:**
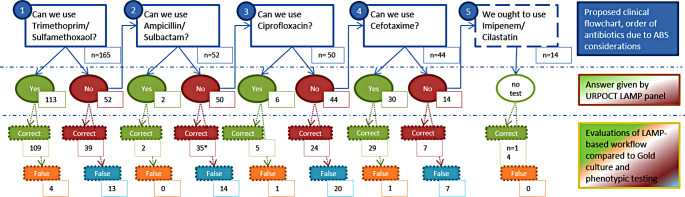
AMS workflow evaluation

Based on the test accuracy data above, we developed a hypothetic algorithm that uses the LAMP resistance panel to inform and improve first line antibiotic selection with AMS aims in mind (antibiotic choice as broad as necessary, but as narrow as possible). We chose five antimicrobials suitable for complicated UTI treatment and sorted them in an order for use following AMS principles. The idea was that one would choose the antibiotic with the narrowest spectrum (“first choice”) unless the LAMP resistance panel would warn about resistance against this – in which case one would default to the second choice antibiotic. If resistance would be detected against the second choice, one would default to the third choice, and so on, and only choose the antibiotic with the broadest spectrum when resistance against the first four options would have been detected. Figure 5 reports how often each antibiotic would have been suggested by this workflow, and how often this would have been correct or incorrect.

## Discussion

Choosing whom to treat for urinary tract infections, and with which antibiotic, is a common conundrum without simple answers. It is also becoming more prominent in populations with a higher percentage of elderly people, and more difficult, as the spread of antimicrobial resistance (AMR) reduces treatment options, especially for initial empiric calculated therapy. EAU recommends the usage of antimicrobial substances for empiric therapy with at least 90% susceptibility of the bacterial species in samples of the relevant patient population. In many settings, this results in prescribing reserve antibiotics. From an antimicrobial stewardship perspective it is clearly necessary to prioritize narrow-spectrum antibiotics like ampicillin-sulbactam, trimethoprim-sulfamethoxazole over broad-spectrum antibiotics such as cefotaxime, piperacillin/tazobactam or carbapenems, to reduce selective pressure. Ciprofloxacin falls somewhere in between, but some phyicians would prefer it due to oral application.

We developed a LAMP panel, suitable for a commercially available machine that can be used in- laboratory or at the point-of-care in a urological ward.

LAMP is a nucleic-acid-amplification technique with parallels to the to PCR technology. Both can be used to detect DNA of bacterial species and resistance genes. Both are theoretically feasible for our approach, we chose to employ LAMP for a couple reasons: First, LAMP reactions run at a constant temperature, whereas PCR needs several heating and cooling cycles. This significantly shortens total detection time. Second, LAMP has been proposed as robust and sensitive (even without complex DNA isolation protocols), which we consider advantageous at the point of care. Third, the machine we run LAMP on is designed for the use at the point of care, and thus easily usable even for non-laboratory-personnel, while also being open to own, custom-made panels.

We focused in this first study on *E.coli*, as the most common uropathogen. Our approach was to first run a detection panel with which to screen multiple samples at once for presence and semi-quantification of *E.coli*, and if positive to test for sensitivities towards small-spectrum antibiotics.

Molecular genetic detection methods are fast compared to culture, but can only target a limited number of genetic targets, thereby standing at risk to falsely declaring bacteria as susceptible if relevant genes are not covered in the test. Due to the genetic diversity being a known trait for *E.coli*, we opted to first characterize the *E.coli* population in the study-relevant urologic patient population.

Our analysis revealed a small group of resistance genes to be present in most isolates resistant to beta lactams and to trimethroprim-sulfamethoxazole. Screening for these genes should therefore allow to rule out resistance in most cases when the respective genes were not found.

For ciprofloxacin, our genomic analysis showed that most sensitive isolates had a full-length hemolysin (*hlyA*) gene, while resistant isolates often had truncated versions or none. This fitted with observations made posthoc in the microbiological laboratory: *E.coli* strains that exhibit β-hemolysis on Columbia Sheep Blood Agar were resistant to fluoroquinolones to a lesser extent (see supplemental Table 4). It is currently unclear if this phenomenon is true in all *E.coli* populations, but was described in several studies from Czech Republic, Spain, and Iran [26–28]. We therefore selected *hlyA* as a marker for ciprofloxacin susceptibility.

Our LAMP primer for resistance detection was designed as follows: *bla*_TEM-1B_ indicates resistance against narrow spectrum beta lactams such as ampicillin/sulbactam and cefuroxime, *bla*_CTX-M-14_ and *bla*_CTX-M-15_ indicates an ESBL phenotype (resistance against ampicillin/sulbactam, cefuroxime, cefotaxime). Both s*ul2* and *dfrA17* indicate resistance against trimethoprim/sulfamethoxazole and absence of “full-length” *hlyA* indicates resistance against ciprofloxacin.

We evaluated our LAMP panel at the point of care in the urological department and prospectively tested samples from newly admitted patients. We took aliquots from 1596 fresh native urine samples for LAMP testing, while the rest of the sample was analyzed in the routine workflows of the clinical microbiology and the clinical chemistry laboratories. Comparison with culture results of the microbiological lab yielded results of 95.4% sensitivity, 96.1% specificity, 79.3% positive predictive value and 99.3% negative predictive value. Subgroup analysis showed no differences in detection when urine was taken from indwelling or disposable catheters (Table 2).

Initially we screened all urine samples without preselection (set 1). Accuracy of 96.36% was good, but with a PPV of 67.27% we had to ensure that our test would not report samples as positive with species other than *E.coli.* Therefore we tested another set of urine samples with an emphasis on culture-positive samples (randomized samples from the same department, but only 10% culture-negative samples): *E.coli* was detected with a comparable accuracy of 95.18%. Lastly, we considered the usage of an automatized preselection: fast urine flow cytometry (UFC) was used to detect significant bacteriuria in the urine samples, and only these samples were further analyzed with LAMP. Accuracy in these samples was again comparable at 95.51%. Thus, LAMP can either be used directly to screen urine samples for *E.coli*, or LAMP can be used as a second step after urine flow cytometry preselection. We demonstrated that time-to-positivity (amplification velocity) of the LAMP reaction was useful in quantifying *E. coli* in patient samples, thus helping in deciding whether “significant bacteriuria”, according to thresholds of ≥ 1 × 10^4^ or ≥ 1 × 10^5^ CFU/ml, is present in the patient samples. Of note, LAMP was more sensitive for the detection of *E. coli*, when compared to culture, and slower amplification curves indicated lower bacterial loads.

Using the visualization in Fig. 4, one could use the timepoint at which the LAMP reaction reaches the threshold of 1 × 10^4^ fluorescence units (TTP, time-to-positivity) to gauge the bacterial loads in the sample: TTP ≤ 9.75 min signal *E.coli* of at least 1 × 10^5^, TTP between 9.75 and 14.75 min signal *E.coli* between 1 × 10^5^ and 1 × 10^4^, TTP between 14.75 and 17 min signal *E.coli* between 1 × 10^4^ nd 1 × 10³ CFU/ml. TTP ≥ 17 min signals *E.coli* < 1 × 10³ CFU/ml, but was also observed in samples with no culturable *E.coli.* Distinguishing these last two cases using the LAMP reaction is not as easy: LAMP reactions from samples with *E.coli* generally showed “typical” sigmoid shaped amplification curves, whereas samples without cultivable *E.coli* often, but not always showed atypical curves, like steadily increasing lines without clear turning point, which nevertheless reaches the fluorescence threshold at similar timepoints. The form of the curve might thus give an indication, but we don’t consider this association strong enough to completely rely on it. We also know that routine culture sometimes misses *E.coli* of 1 × 10³ CFU/ml. As both routine culture and LAMP seem to be reaching their respective limits with these low concentrations, results need to be interpreted with caution.

In regard to false positive LAMP screening, it is possible that LAMP did correctly detect *E.coli* DNA within the sample, but the *E.coli* cells were not viable for culture. We speculate that such could be the case due to antibiotic therapy administered prior to sampling, or due to patients’ immune response.

False negative results could theoretically result from the strains in question displaying mutations within the target gene which would inhibit LAMP detection. In rare cases it could be possible that the strains were actually misidentified as *E.coli* in routine diagnostic, but we consider this a rather low priority, as every identification was performed using MALDI-TOF which is considered very accurate, especially in identifying *E.coli* species. In either case, pipetting errors can never be completely ruled out, as the LAMP assay was done as a point of care study, such as to emulate a real patient care setting.

All samples positive for *E.coli* where subsequently tested with our resistance panel. Our panel excelled at predicting susceptibility of the isolates to ampicillin, ampicillin/sulbactam, cefuroxime, cefotaxime, piperacillin/tazobactam, ceftazidime, trimethoprime/sulfamethoxazole and ciprofloxacin (negative predictive values of 92.8%, 96.4%, 92.8%, 97.8%, 97.8%, 97.8%, 96.5% and 96.8%, respectively). Positive predictive values (predictors for resistance) were lower in general (89.6%, 72.7%, 26.0%, 68.2%, 31.8%, 45.5%, 81.3%, 29.6%, respectively). False negative results could result from resistance genes that were not part of our panel. The LAMP system we used only allows for 8 reactions at a time, which limited the target gene spectrum to be tested in one run. Therefore, it was to be expected that some resistances can be missed by design with a purely genetic screening approach using a limited number of targets. The feasibility of using such a system is therefore dependent on the population it is used for: in our patient population, these accuracy data were sufficient to reach high negative predictive values – which means that recommendations can be drawn without compromising patient safety. Our panel screened for *bla*_TEM−1B_, *bla*_CTX−M−14_ and *bla*_CTX−M−15_^,^ which are common in our population. In populations where resistance to β-lactams is conferred more frequently by genes such as *bla*_SHV−12,_*bla*_CTX−M−1,_ or *ampC-*type genes or other genes instead, our panel would possibly be impaired and had to be adapted to the local situation [29].

False positive results could result from strains that do not express their respective resistance genes, or it could be due to resistant strains missed during routine testing: for cultural resistance testing, colonies are from an agar plate. Even though colonies may appear identical phenotypically, *E.coli* with various subpopulations and with different resistance profiles can be present (e.g. hetero-resistance). In fact, when examining the patient history for false negative cases, we found that in several of those cases, patients would later present with *E.coli* strains that showed the resistance profiles predicted previously by LAMP, the growth of which was not recognized in the initial culture that was compared with the respective LAMP result.

We follow with the idea of Diagnostic Stewardship to use the right test for the right patient at the right time. Asymptomatic patients usually don’t need to be tested or treated, female uncomplicated UTI patients typically can receive empirically calculated nitrofurantoin, fosfomycin or pivmecillinam, and critically ill patients with urosepsis may need reserve broad spectrum antibiotics. There are, however, a huge number of patients with complicated UTI who fall in between: some of which could benefit from more narrow spectrum antibiotics like ampicillin/sulbactam or trimethoprim/sulfamethoxazole, but due to high AMR rates in the population physicians are compelled to use more broad spectrum antibiotics like third generation cephalosporins or piperacillin/tazobactam instead. Our LAMP approach aims to enable physicians to give the right antibiotic to the right patient in these situations.

To limit treatment failures, EAU recommends to only use antibiotics with at least 90% susceptibility in this situation (i.e. complicated lower UTIs) [24], which in many settings means ampicillin/sulbactam, trimethoprim/sulfamethoxazole and ciprofloxacin are excluded from initial therapy. Our goal is to predict susceptibility of these antibiotics with at least 90% certainty (negative predictive value). Within our patient population, we met this threshold with predictive values of 92.8 to 97.8%. This means that using the LAMP panel to screen for *E.coli* resistance would re-enable usage of these antibiotics in this setting.

This is in line with a key clinical demand of AMS principles: to use rapid and reliable diagnostics for calculated antibiotic therapy, thus saving the use of broad spectrum antibiotics for cases when deemed necessary. To quantify this potential benefit of our LAMP panel for clinical use, we developed an example algorithm for LAMP-guided antibiotic prescription. Five antibiotics were ordered from most preferable to administer (first line), to least preferable (last resort), following AMS principles:

1. Trimethoprim/sulfamethoxazol.
2. Ampicillin/sulbactam.
3. Ciprofloxacin.
4. Cefotaxime.
5. Imipenem.

The LAMP results would inform whether an antibiotic could be used or not, with the preference given to the lowest number on the list that LAMP did not detect any resistance against (see Fig. 4). Following this algorithm a clinician would prescribe trimethoprime/sulfamethoxazole to 113 patients, ampicillin/sulbactam to 2 patients, ciprofloxacin to 6 patients, cefotaxime to 30 patients and imipenem/cilastatin to 14 patients. The majority of these recommendations would be considered appropriate and correct when compared to classical phenotypical resistance testing (only 6 of 165 patients would have received an antibiotic against which phenotypic resistance was detected).

This proof-of-concept study was focused on *E. coli*, because it is the most common bacterial uropathogen and shows a high variability in terms of antibiotic resistance profiles. An advantage of our approach is the usage of an already commercially available machine, where we needed to supply custom tailored primer setups to screen for *E.coli* and the chosen resistance parameters. For clinical use however, we would aim to expand the panel to include more of the commonly encountered uropathogens, most importantly other Enterobacterales, *Pseudomonas aeruginosa* and Enterococci. Also, it is feasible to include more resistance genes to further improve safety (which might require using more 8-strips at once). The potential to provide meaningful information to clinicians is supported by other studies using a molecular approach for resistance prediction (26). Our study shows that clinically significant data can be retrieved by a simple sub-genomic based assay (a LAMP panel). Nevertheless, the data presented is based on a single site testing for which the panel was designed and needs to be broadly evaluated for general application – it is possible that different sites require different genetic resistance targets.

In summary, the LAMP panel described in this study can rapidly predict antibiotic sensitivity in UTIs caused by *E. coli* in urologic patients presenting at a hospital department of urology. As a future perspective, the large-scale application of such an approach offers the potential to limit empirical antibiotic overtreatment of UTIs and is thereby an instrument enabling reduction in resistance-promoting selective pressures of antibiotic usage in medicine.

## Conclusions

Our proof of concept study demonstrates how the implementation of a custom-tailored LAMP panel can provide detection, quantification and antibiotic susceptibility results for *E. coli* in urine samples of urological patients in about one hour at the point of care. This approach is promising to improve initial calculated antimicrobial treatment by providing rapid results at the point of care and has the potential to limit broad-spectrum antibiotic overuse, thereby assisting antimicrobial stewardship efforts.

## Electronic supplementary material

Below is the link to the electronic supplementary material.

Supplementary Material 1

Supplementary Material 2

Supplementary Material 3

Supplementary Material 4

Supplementary Material 5

Supplementary Material 6

Supplementary Material 7

## Data Availability

No datasets were generated or analysed during the current study.
