# Supplementary figures and images for: Genome-based development and clinical evaluation of a customized LAMP panel to rapidly detect, quantify, and determine antibiotic sensitivity of Escherichia coli in native urine samples from urological patients
Source: Eur J Clin Microbiol Infect Dis. 2025 Jan 7;44(3):703–15. doi: 10.1007/s10096-024-05030-3 (PMC11880174; doi:10.1007/s10096-024-05030-3)

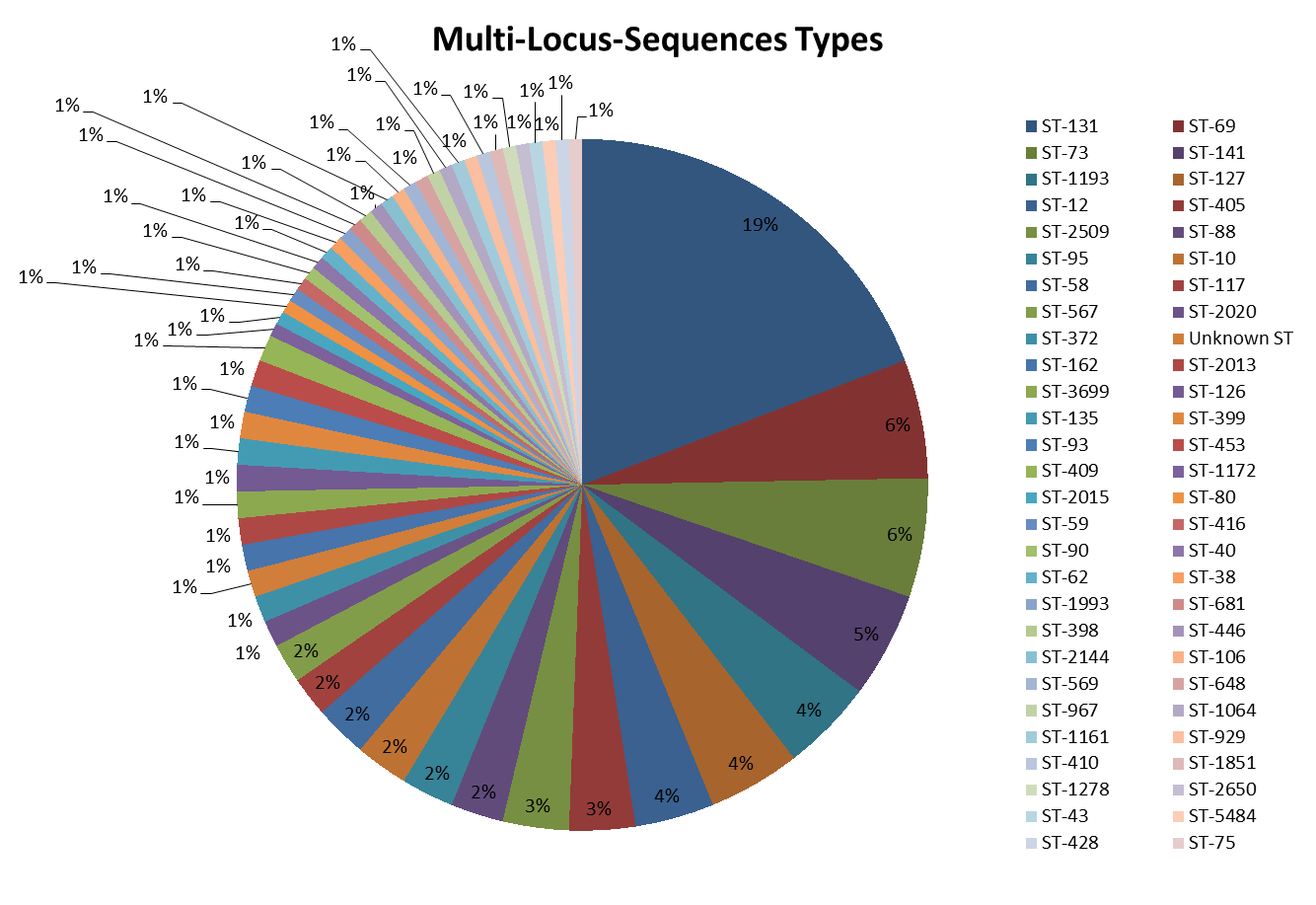

Supplement: Supplementary file 1 — Supplementary Material 1 [file 10096_2024_5030_MOESM1_ESM.png]

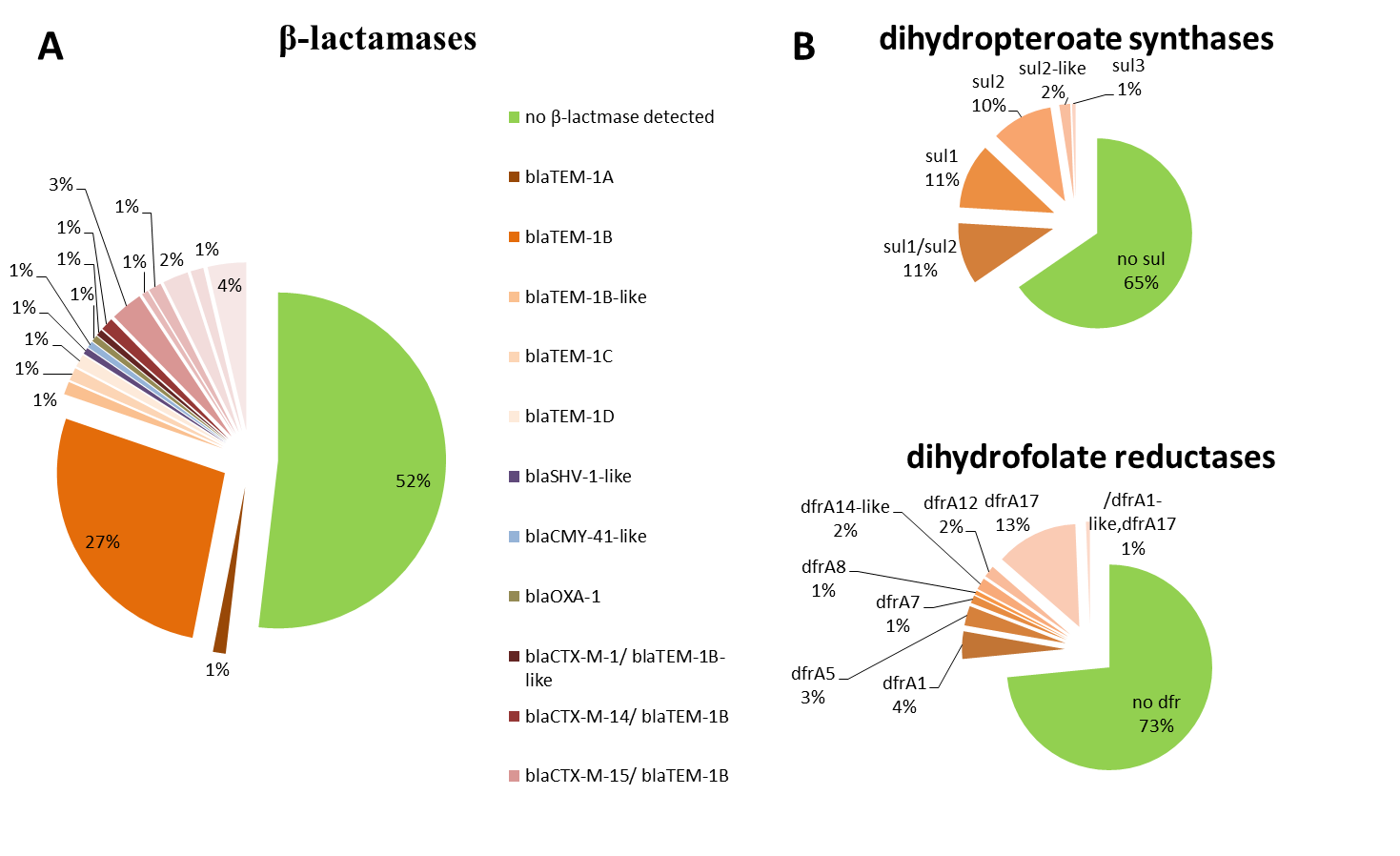

Supplement: Supplementary file 2 — Supplementary Material 2 [file 10096_2024_5030_MOESM2_ESM.png]

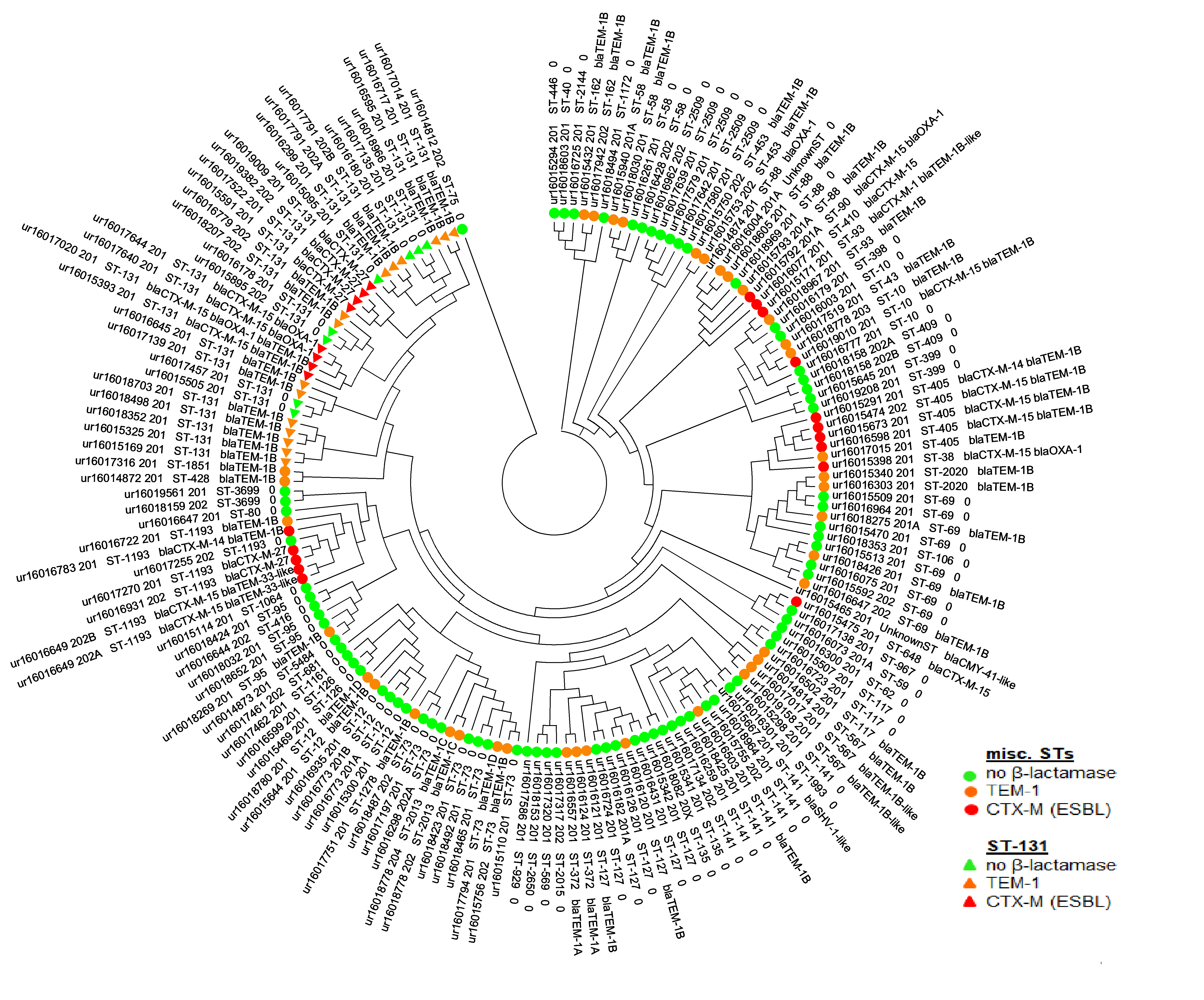

Supplement: Supplementary file 4 — Supplementary Material 4 [file 10096_2024_5030_MOESM4_ESM.png]
