# Supplementary material for: Genome-based development and clinical evaluation of a customized LAMP panel to rapidly detect, quantify, and determine antibiotic sensitivity of Escherichia coli in native urine samples from urological patients
Source: Eur J Clin Microbiol Infect Dis. 2025 Jan 7;44(3):703–15. doi: 10.1007/s10096-024-05030-3 (PMC11880174; doi:10.1007/s10096-024-05030-3)
Supplement: Supplementary file 6 — Supplementary Material 6 [file 10096_2024_5030_MOESM6_ESM.docx]

| **Supplemental Table 4A: Hemolysin A Genotype vs. ciprofloxacin resistance** | | | **Size of CDS „*hlyA*“** | |  | **Supplemental Table 4B: Phenotypic hemolysis on Columbia Sheep Blood Agar vs. ciprofloxacin resistance** | | **Phenotypic hemolysis on Columbia blood agar** | |
| --- | --- | --- | --- | --- | --- | --- | --- | --- | --- |
|  |  |  | >=2400 bp | <2400bp or none |  |  |  | β-hemolyis | γ-hemolysis |
|  | Total | 162 | 39 | 123 |  | total | 479 | 82 | 397 |
| **Ciprofloxacin**  **Resistance** | sensitive | 110 | 35 | 75 |  | sensitive | 357 | 72 | 285 |
|  | resistant | 52 | 4 | 48 |  | resistant | 122 | 10 | 112 |

Supplemental Table 4A: Analysis of the 162 reference *E.coli* isolates: “full length” *hlyA* in the isolates’ genomes was associated with a lower rate of phenotypic ciprofloxacin resistance

Supplemental Table 4B: To quantify the experience that β-hemolyis was associated with less resistance, we proceeded to note β-hemolyis on prospective *E.coli* isolates in urine samples in the microbiological laboratory in general (i.e. not samples and strains specific for this study, but independent prospective samples). As was surmised, strains with β-hemolyis were resistant less often.
